# Underdiagnosis of malnutrition in infants and young children in Rwanda: implications for attainment of the Millennium Development Goal to end poverty and hunger

**DOI:** 10.1186/1475-9276-10-61

**Published:** 2011-12-29

**Authors:** Agnès Binagwaho, Mawuena Agbonyitor, Alphonse Rukundo, Niloo Ratnayake, Fidel Ngabo, Josephine Kayumba, Bridget Dowdle, Elena Chopyak, Mary C Smith Fawzi

**Affiliations:** 1 Government of Rwanda, Ministry of Health, P.O. Box 3622, Kigali, Rwanda; 2 National University of Rwanda, P.O. Box, 56 Butare, Rwanda; 3 Department of Global Health and Social Medicine, Harvard Medical School, 641 Huntington Ave., Boston, MA 02115, USA; 4 Government of Rwanda, Ministry of Health, Malaria Unit/TRAC Plus, P.O. Box 2717, Kigali, Rwanda; 5 University of Virginia, P.O. Box 800793, Health Sciences Center, School of Medicine, Charlottesville, VA, USA; 6 Government of Rwanda, Treatment and Research AIDS Center (TRAC) P.O. Box 2717, Kigali, Rwanda; 7 Partners In Health, 888 Commonwealth Ave., Boston, MA 02215, USA

**Keywords:** child malnutrition, growth standards, Rwanda, diagnosis

## Abstract

Progress towards the first Millennium Development Goal (MDG1) to end poverty and hunger has lagged behind attainment of other MDGs due to chronic poverty and worldwide inequity in access to adequate health care, food, clean water, and sanitation. Despite ongoing challenges, Rwanda has experienced economic progress and the expansion of the national public health system during the past 20 years. However, protein-energy malnutrition in children under five is still a major concern for physicians and government officials in Rwanda. Approximately 45% of children under the age of five in Rwanda suffer from chronic malnutrition, and one in four is undernourished. For years, health facilities in Rwanda have used incorrect growth references for measuring nutritional status of children despite the adoption of new standards by the World Health Organization in 2006. Under incorrect growth references used in Rwanda, a number of children under five who were severely underweight were not identified, and therefore were not treated for malnutrition, thus potentially contributing to the under five mortality rate. Given that one in ten children suffer from malnutrition worldwide, it is imperative that all countries with a burden of malnutrition adopt the most up-to-date international standards for measuring malnutrition, and that the problem is brought to the forefront of international public health initiatives. For low income countries in the process of improving economic conditions, as Rwanda is, increasing the identification and treatment of malnutrition can promote the advancement of MDG1 as well as physical and cognitive development in children, which is imperative for advancing future economic progress.

## Introduction

Over three million deaths occur from protein-energy malnutrition (PEM) in children under five worldwide annually [1]. The burden of malnutrition in the developing world accounts for a large majority of these deaths, where one in four children under five are underweight. Based on this level of malnutrition it is unlikely that the MDG1 goal of eradicating extreme poverty and hunger will be attained by the year 2015 [2]. In Rwanda, an estimated 45% of children under five suffer from chronic malnutrition [3]. The Ministry of Health estimates malnutrition as one of the ten leading causes of death for children under age five, with hidden or unreported malnutrition contributing to more than half of child deaths [4]. The substantial burden of infectious disease contributes to the high rate of malnutrition among children, leading to negative effects on growth, as well as increased vulnerability to future occurrences of infection. Improved nutrition can reduce child morbidity and mortality related to infectious disease, as adequately nourished children are more likely to fight and recover from an infection, to avoid the negative effects of the infection on growth, and to resist the repeated occurrence of infection [5].

Due to the high prevalence of malnutrition in children under five and the implications for morbidity and mortality, it is essential that health professionals and paraprofessionals have access to the best tools possible for determining a child's general nutritional status in community, health center and hospital settings. This paper examines the growth references used prior to 2009 in Rwanda to assess nutritional status of children under the age of five, and outlines the problem of the limited capacity to identify malnourished children due to inadequate technical input by national and multinational experts. In addition, the paper describes the process in which the growth references used in Rwanda were updated to correspond with the international standards developed by WHO in 2006 [6].

## Discussion

### Background on references and standards to assess child growth and malnutrition

In 1977, the United States National Center for Health Statistics (NCHS) produced growth references to assess proper development. The growth references used weight-for-age, height-for-age and weight-for-height measurements to identify underweight, stunted, wasted, and obese children. These charts based on the NCHS reference population were distributed throughout the United States and normalized versions were used by the World Health Organization (WHO) to develop international growth references [7]. By 1998 a number of studies had demonstrated that these international growth references were inadequate, specifically for breast-fed infants [8,9]. This was due in part to questions that arose regarding the methods used to determine the original NCHS growth references, which were based on primarily formula fed middle-class infants living in the U.S. (Ohio) between 1929 and 1975.

In 2006, 29 years after the original NCHS growth references were published, and following eight years of debate and new research, the WHO released new child growth standards [6]. The 2006 WHO growth charts were based on primary data collected from selected sites in Brazil, Ghana, India, Norway, Oman and the U.S. from 1997 to 2003. Although the new WHO charts were not based on a purely representative sample of children under five worldwide, the broader range of sites indicates that the new charts are more applicable for different populations as compared to the NCHS growth references. In addition, the WHO sample population included only breast-fed infants and included measurements of infant weight and length taken longitudinally every two weeks for the first two months and monthly thereafter (compared to the data that were collected cross-sectionally for the NCHS reference populations) [10]. In a direct comparison of the new WHO standards and the NCHS/WHO growth references, de Onis et al. (2006) observed the prevalence of severe wasting using the new WHO standards as 1.5 to 2.5 times greater than the prevalence based on the NCHS/WHO growth references. Therefore, there are significant practical implications of appropriately using the new WHO child growth standards to identify and treat children with severe malnutrition; whereby using the old references would result in missing children in need of acute care [10].

### History of rwandan national guidelines for malnutrition

Before May 2009, health facilities in Rwanda used graphs in a booklet of children's health published in 1978 and entitled *Ifishi Y'Ubuzima Bw'umwana (Child Health Card) *as the growth references for measuring malnutrition. The graphs, or *Imikurire Y'Umwana (Child Growth) *were used to track children's growth based on weight-for-age. The graph is used for the purpose of detecting and monitoring malnutrition for all Rwandan children under five years of age in Rwanda. The booklet was given to all caregivers of children in Rwanda at the time of first DPT vaccination (DPT1) of the child in government as well as non-government clinics throughout the country; prior to the development of the new guidelines coverage for the DPT1 was 96% in 2007 [11]. Parents were instructed to bring the booklet to subsequent vaccination appointments so that weight-for-age could be measured regularly and malnutrition could be readily identified and treated.

Between September 2008 and January 2009 a comprehensive desk review was conducted with analyses of reports and policies and interviews with professionals in the Rwandan health sector. This assessment did not reveal the origin of the design of the *Ifishi Y'Ubuzima Bw'umwana *growth chart. Furthermore, the growth chart did not rely on current international standards or previous growth references of malnutrition. Meanwhile, the 2006 WHO growth charts were available for use internationally.

**Figure 1:**
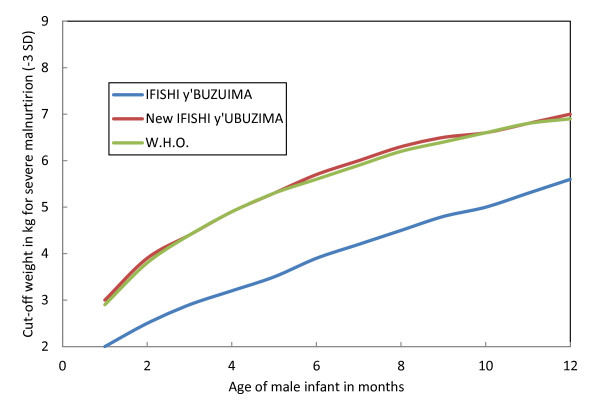
**Comparison of weight-for-age charts of infants under 1 year**.

As a result of the design of the Rwandan growth charts used before May 2009, malnourished children were underdiagnosed and undertreated. For example, a six-month-old boy weighing 4 kilograms would be classified as severely malnourished under the 2006 WHO growth standards and require hospital admission given the high risk of mortality. Using *Ifishi Y'Ubuzima Bw'umwana*, this same child would be classified as being of normal weight would therefore be sent home without further assessment or treatment (see Figure 1).

**Figure 2:**
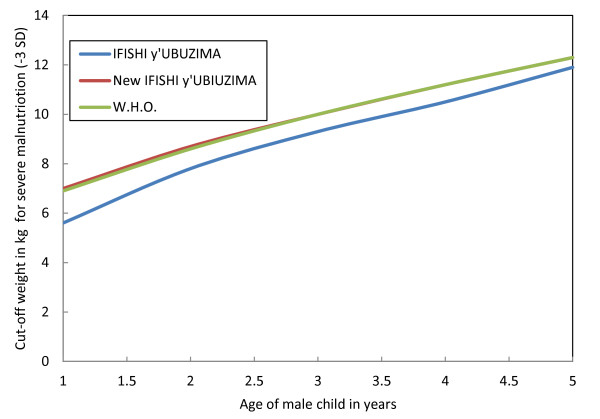
**Comparison of weight-for-age charts for children from 1-5 years**.

Unfortunately, nutritional assessments based on the *Imikurire Y'Umwana *booklet were also inaccurate for children one to five years of age. The *Imikurire Y'Umwana *chart underestimated children's growth from birth until 59 months of age. Children from one to five years old may have been severely malnourished but were instead classified as moderately malnourished, thereby delaying the life-saving treatment they needed at a health facility. Figure 2 illustrates this point as it shows that children with the same weight-for-age may be considered severely malnourished on the 2006 WHO chart, and normal or only moderately malnourished on the *Imikurire Y'Umwana *graph.

### What has been done to address the situation

Between 2006 and 2009, following the release of the new WHO standards, UN agencies in Rwanda continued to fund the duplication, dissemination and training in the use of *Imikurire Y'Umwana*, an inaccurate growth monitoring chart. This occurred despite representation of the Ministry of Health's Technical Working Group on Nutrition (TWG) within UN agencies. The TWG was created to advise the Ministry of Health and had full access to all information related to the "rolling out" of the new WHO growth standards.

The movement to correct the tools and guidelines came from outside the TWG and was initiated by the Rwandan Pediatric Society as late as 2008 and revealed the discrepancies between the 2006 WHO standard growth chart and the *Imikurire Y'Umwana *growth chart and the serious consequences. In response, beginning in early 2009, the Ministry of Health initiated the process of changing the national growth chart, guidelines and protocols to correspond with the 2006 WHO growth standards in order to improve the diagnosis of undernutrition and the care of malnourished children under five years of age in Rwanda.

The Rwandan Ministry of Health employed guidelines from the WHO using the updated 2006 child growth standards [6] and the *"*Management of severe malnutrition,*" *a manual for physicians and other senior health workers, [12] as substantial sources in developing the new national protocols for managing malnutrition. The new document described underweight children under five years of age as being two standard deviations below their expected weight-for-age based on updated international growth standards issued in 2006. Chronic malnutrition (stunting) for children under five years of age was described as being two standard deviations below the expected height-for-age. Wasting for children under five years of age was defined as two standard deviations below the expected weight-for-height according to the new WHO international growth standards [13,14].

**Figure 3:**
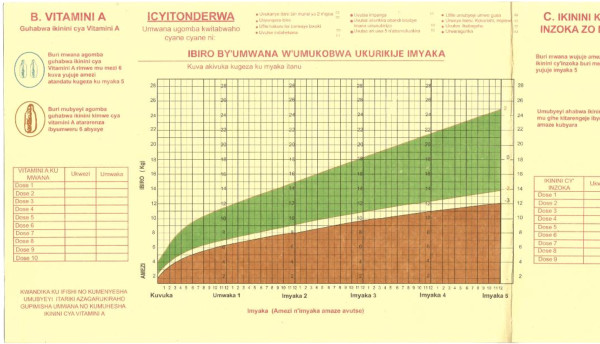
**IBIRO BY'UMWANA W'UMUKOBWA UKURIKIJE IMYAKA - The new and correct growth chart for girls currently used to monitor malnutrition in Rwanda**.

**Figure 4:**
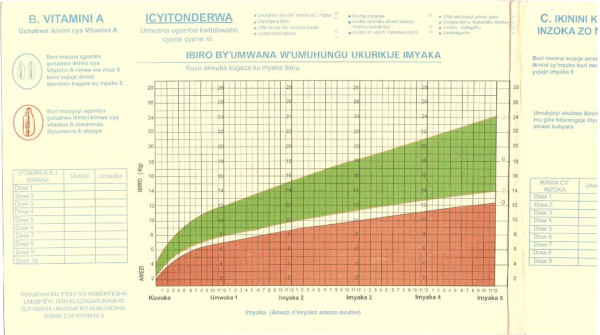
**IBIRO BY'UMWANA W'UMUHUNGU UKURIKIJE IMYAKA - The new and correct growth chart for boys currently used to monitor malnutrition in Rwanda**.

The new national growth charts (Figures 3 and 4) were finalized in early April 2009, then produced and distributed in May 2009. The new charts are used in all health facilities in Rwanda and are available on the Internet in addition to hard copy form at 426 health centers and 42 district hospitals [15].

The example from Rwanda is not isolated, and may be indicative of similar evaluation problems in other countries. Through a study conducted in a malnutrition treatment program in Maradi, Niger, it was demonstrated that the revised growth standards and cut-off points for malnutrition under the updated 2006 WHO guidelines offered more sensitive and accurate assessment, which were related to shorter duration of treatment and lower mortality rates for malnourished children [16]. In order to assure accuracy, particularly when new international guidelines and protocols are brought forward for national adoption, the technical agenda of international organizations (WHO, UNICEF, UNFPA), should prioritize an annual review of accuracy of tools and guidelines in high burden countries.

Utilizing accurate and updated measurement standards can have far-reaching effects that transcend the immediate indicators observed. As nutrition is linked to physical and cognitive growth, as well as to the management and progression of some infectious diseases such as TB and HIV, the updated standards have the potential to increase the identification of children suffering from infection. This may lead to earlier detection of TB and/or HIV and better survival rates and outcomes particularly for children who are found to be acutely malnourished, since TB is difficult to diagnose in children [17]. As higher numbers of children in Rwanda are now more likely to be treated for malnutrition, those also suffering from TB and HIV will also benefit, therefore promoting better health overall. Malnutrition and infectious disease in a child under five years of age can have a negative lifetime affect on his/her emotional and intellectual development [18]. At the societal level, malnutrition can inhibit a country's long-term economic development since economic growth is contingent upon a healthy and productive workforce [19]. The role of the health of the Rwandan population will become increasingly apparent as national officials strive to sustain recent levels of economic growth.

## Conclusions

The use of outdated tools, such as the *Imikurire Y'Umwana *booklet, which lack transparency concerning the criteria used in determining nutritional standards, has led to the mismanagement of nutritional status and incorrect follow-up of undernourished children under five in Rwanda prior to May 2009. Additionally, the insufficient guidelines may have contributed to the lack of progress despite a decades-long fight against malnutrition in Rwanda. Although there can be a range of reasons that malnutrition is not adequately identified and treated, such as difficulty in ascertaining the age of the child as well as lack of access to treatment, it remains that the issue of undetected and untreated malnutrition in children under five due to the use of inappropriate growth references may have resulted in preventable morbidity and mortality in this vulnerable population, particularly for children suffering from severe acute malnutrition.

National technicians and experts should periodically review local protocols and tools for appropriateness, and should be more vigilant in tracking the evolution of global standards, rapidly making required adjustments and ensuring they are implemented. The delay in adopting accurate measures of malnutrition and the subsequent ramifications point to the need not only for national working groups to remain abreast of new information, but also for greater vigilance on the part of the UN to ensure that any change of standards affecting common health practices globally is fully disseminated in a timely fashion to developing countries through their country offices. Ensuring the appropriate updating of growth standards in resource-poor contexts similar to Rwanda may facilitate progress towards MDG1 which has lost ground in recent years due in part to the global economic crisis and the chronic inequity in access to food as well as health and other basic services [2].

## Abbreviations

CDC: Centers for Disease Control and Prevention; HIV: Human immunodeficiency virus; PEM: Protein-energy malnutrition; TWG: Technical Working Group on Nutrition; TB: Tuberculosis; UNICEF: United Nations Children's Fund; UNFPA: United Nations Population Fund; NCHS: United States National Center for Health Statistics; WHO: World Health Organization.

## Competing interests

The authors declare that they have no competing interests.

## Authors' contributions

AB is responsible for the primary conceptualization, drafting, and direction of the manuscript, and MCSF is responsible for coordinating the review and drafting the final manuscript. All of the authors listed have been involved in the logistics and drafting of the manuscript, which warrant co-authorship. The international coordination of this manuscript required a team with access to a variety of sources of information and tools. The manuscript was read and approved by all authors.

## Author's information

Name Abbreviation, Title, Institutions, Country

*A. Binagwaho*: Minister of Health, Government of Rwanda, Ministry of Health, Rwanda,

*M. Agbonyitor*: Research Assistant, Department of Global Health and Social Medicine, Harvard Medical School

*A. Rukundo*: Monitoring and Evaluation Officer, Government of Rwanda, Ministry of Health, Malaria Unit/TRAC Plus, Rwanda

*B. Dowdle*: Partners In Health, USA

*N. Ratnayake*: University of Virginia, USA

*F. Ngabo*: Family Planning/Maternal and Child Health Team Leader, Government of Rwanda, Ministry of Health, Rwanda

*J. Kayumba: *Nutrition and HIV/AIDS Officer, Treatment and Research AIDSCenter (TRAC), Rwanda,

*M.C. Smith Fawzi: *ScD, Epidemiologist, Partners In Health, and Instructor, Department of Global Health and Social Medicine, Harvard Medical School, USA

*E. Chopyak: *Research Assistant, Department of Global Health and Social Medicine, Harvard Medical School/Harvard School of Public Health, USA
